# Risk of Second Primary Cancers Among Long-Term Survivors of Breast Cancer

**DOI:** 10.3389/fonc.2019.01426

**Published:** 2020-01-13

**Authors:** Dan Li, Shanshan Weng, Chenhan Zhong, Xiujun Tang, Ning Zhu, Yi Cheng, Dong Xu, Ying Yuan

**Affiliations:** ^1^Department of Medical Oncology, The Second Affiliated Hospital, Zhejiang University School of Medicine, Hangzhou, China; ^2^Department of Surgical Oncology, The Second Affiliated Hospital, Zhejiang University School of Medicine, Hangzhou, China; ^3^Key Laboratory of Cancer Prevention and Intervention, China National Ministry of Education, Key Laboratory of Molecular Biology in Medical Sciences, Cancer Institute, The Second Affiliated Hospital of Zhejiang University School of Medicine, Hangzhou, China

**Keywords:** cancer risk factor, risk model, survival, breast cancer, second primary cancers

## Abstract

**Purpose:** The current study explored the risk of developing second primary cancers (SPCs) among long-term early-stage breast cancer survivors and identified risk factors to build an externally validated clinical prediction model.

**Methods:** The cumulative incidence of SPCs was calculated by Gray method among survivors of early-stage initial primary breast cancer (IPBC). Comparisons of treatment-related risk by selected organ sites were performed. A nomogram was established to estimate the individual risk of developing SPCs based on the multivariate Fine and Gray risk model. Decision curve analysis (DCA) was used to evaluate clinical usefulness of the model.

**Results:** The cumulative incidence of developing SPCs after early-stage IPBC was 7.43% at 10 years, 14.41% at 15 years, and 20.08% at 20 years. Radiotherapy was associated with elevated risks of any SPCs and with elevated risks of lung cancer (SHR: 1.109; *P* = 0.045), breast cancer (SHR: 1.389; *P* < 0.001), and AML (SHR: 1.298; *P* = 0.045). Chemotherapy was significantly associated with a declined risk of any SPCs, with decreased risks of lung (SHR: 0.895; *P* = 0.015) and breast cancers (SHR: 0.891; *P* < 0.001), as well as elevated risks of other leukemias (SHR: 1.408; *P* = 0.002). HR-positive status was associated with decreased risks of any SPCs; with decreased risks of breast (SHR: 0.842; *P* < 0.001) and ovarian cancers (SHR: 0.483; *P* < 0.001); and with elevated risks of urinary tract cancers (SHR: 1.214; *P* = 0.029).

**Conclusion:** We found that the cumulative incidence of developing SPCs increased over time and did not plateau. Risk factors for developing SPCs identified by our study were not consistent with those of previous studies. The prediction model can help identify individuals at higher risk of SPCs.

## Introduction

Breast cancer is worldwide the leading cancer among women (1). Advances in early systematic screening, effective treatments, and supportive care have caused an elevated proportion of breast cancer survivors (2). For some survivors, these survival benefit have been diluted by the late long-term effects of initial cancer and its therapy, with second primary cancers (SPCs) comprising one of the most potentially life-threatening sequelae (3). Previous population-based researches have examined the risk of developing SPCs among initial primary breast cancer (IPBC) survivors compared to the general population. However, the results from these studies were inconsistent in risk estimation, with an elevated risk range from 15 to 45% for any types of SPCs (2). It is difficult to find an exact estimation of how frequently SPCs occur or the likelihood that IPBC survivors will develop one (4).

Risk stratification by age and race have been extensively explored, demonstrating that survivors of premenopausal age at initial diagnosis and black women had an elevated risk of developing SPCs (2). Each of these previously used methods has inherent limitations when attempting to ascribe causation, especially when several risk factors are involved (5). Therefore, the patterns of SPC development are still poorly understood. Clinicopathological factors have also been proposed to explain the elevated risks. Only a few researches estimated the effect of initial treatment on the development of SPCs (2, 6). The results from these researches were inconsistent, and prediction models of developing SPCs were not provided for survivors.

The purpose of the current research is to estimate cumulative incidence of SPCs and examine risk factors of developing SPCs in long-term early-stage breast cancer survivors in the presence of competing risks. Furthermore, we built an externally validated competing nomogram to help select patients at increased risk of developing SPCs.

## Methods

### Inclusion and Exclusion Criteria

Only Female breast cancer patients in the National Cancer Institute Surveillance, Epidemiology, and End Results (SEER) registry with histologically confirmed early-stage (stage I–III) who survived for 5 years and more were retrospectively reviewed from 1990 to 2010. In total, 250,764 eligible female patients at 20–80 years old with complete clinicopathological information were included. The inclusion and exclusion criteria was showed in flow chart (Supplemental Figure 1). The follow-up time for SPCs for each patients began 5 years after the IPBC diagnosis and ended at diagnosis of SPCs, death from IPBC or the end of follow up (December 2017), or death from other causes.

### Variable Declaration

Age was regrouped into four subpopulation (20–40, 41–60, 61–70, and 71–80). Race was regrouped into white race, black race and other race. Marital status was regrouped into married status, single status or divorced status. The hormone receptor (HR) status was stratified to HR positive (estrogen receptor (ER) or progesterone receptor (PR) was positive) and HR negative (both ER and PR were negative). Histology was divided as invasive ductal carcinoma (IDC), invasive lobular carcinoma (ILC), mixed (mix of IDC and ILC), and other. Surgery was regrouped as breast conserving surgery (BCS) (including partial mastectomy, lumpectomy excisional biopsy, and segmental mastectomy) and mastectomy (including total mastectomy, modified radical mastectomy, radical mastectomy, extended radical mastectomy). Topography and morphology were used to explore the organ site specific risk using International Classification of Diseases for Oncology (ICD-O).

### Study Design and Methods

The cumulative incidence of SPCs was calculated based on the Gray method with a competing risk framework: deaths from IPBC or other causes, whichever occurred first, was regarded as competing event (7). The Kaplan-Meier method was constructed to estimate difference in overall survival (OS) between survivors with and without SPCs.

We randomly divided the entire cohort into a development cohort (75%) and another validation cohort (25%) for development and validation of the competing risks nomogram. Standardized mean differences (SMDs) was used to assess distributional differences in the baseline variables between the development and validation cohorts. Values of *P* > 0.1 imply a potential difference between development and validation cohort (8).

### Variable Selection

The forward and backward stepwise methods was used to select the predictive variables from the development cohort for the prediction model based on the Akaike information criterion (AIC) (9). To further reduce the final model, multivariate Fine and Gray competing risks regression model was used to exclude variables based on a backward selection algorithm with a *P* > 0.05. Furthermore, based on all of the selected features, independent effects of initial cancer treatment (chemotherapy and radiotherapy) and HR status on the risk of developing SPCs in selected organ sites were also examined based on the multivariable competing risk model (7).

### Validation of the Prediction Model

We assessed the calibration for risks of developing SPCs by comparing the observed risks based on the Gray method with the mean predicted risks predicted risks from the prediction model. Likewise, an external validation was performed in the validation cohort. The C-index was also used to quantify the discrimination ability of the prediction model.

### Risk Stratification Ability

The decision curve analysis (DCA) in the validation cohort was used to examine the clinical utility and net benefits of competing risks model for developing SPCs. DCA is a suitable method for evaluating alternative diagnostic and prognostic strategies that has advantages over other commonly used measures and techniques (10). We divided the survivors into three subgroups by the 25th and 75th percentile risk score of the nomogram-based estimated SPC risks. We then calculated the cumulative incidence using the Gray method for each subgroup and compared them across the different risk subgroups (7).

All statistical analysis were conducted using R software (https://www.r-project.org/). Significance level set as *P* < 0.05.

## Results

### Patient Characteristics

**Figure 1:**
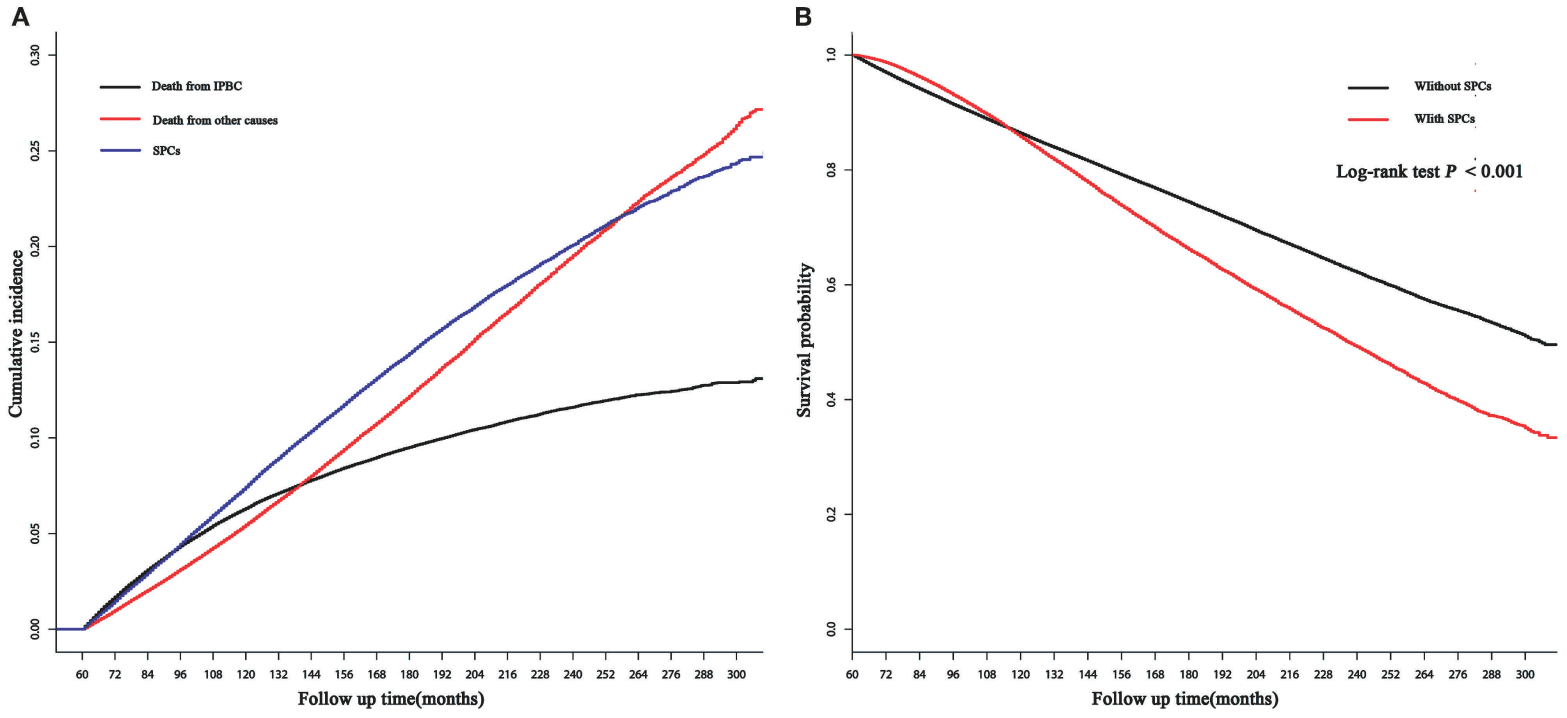
**(A)** Cumulative incidence of second primary cancers (SPCs), death from initial primary breast cancer (IPBC), and death from other causes in the entire cohort based on the Gray method; **(B)** overall survival (OS) between survivors with and without SPCs in the entire cohort based on the Kaplan-Meier method.

250,764 early-stage IPBC patients who survived >5 years between 1990 and 2010 were included in the entire cohort. Of those patients, 30,285 (12.08%) patients developed SPCs. Twenty thousand one hundred and seventy (8.04%) patients died from IPBC, and 26,572 (10.60%) died from other causes. Second breast cancers represented 13,105 (43.27%) of all SPCs followed by gastrointestinal (GI) at 4,325 (14.28%), lung at 3,203 (10.58%), female genital tract at 2,923 (9.65%), skin at 1,467 (4.84%), central nervous system at 1,333 (4.40%), leukemias at 1,253 (4.14%), urinary tract at 1,192 (3.94%), and lymphoma 546 at (1.80%). The median latency from diagnosis of IPBC to subsequent diagnosis of SPCs was 116 months (25–75% interquartile range, 86–153 months). The detailed information of population is summarized in Table 1. In the entire cohort, cumulative incidence of SPCs, in the presence of competing risks of death, was 7.43, 14.41, 20.08% at 10, 15, and 20 years (Figure 1A). There is a significant difference in OS between survivors with and without SPCs (85.77 vs. 86.37% at 10 years, 66.16 vs. 74.39% at 15 years, 49.21 vs. 62.16% at 20 years, *P* < 0.001; Figure 1B).

**Table 1:** Patient characteristics and clinicopathological variables with stratified events.

**Risk factors**		**Stratified events, no. (%)**			
	**Total**	**Censored**	**Death from IPBC**	**Death from other causes**	**SPCs**
	**(250,764)**	**(173,737)**	**(20,170)**	**(26,572)**	**(3,0285)**
**Age**					
20–40	23,537	18,316 (10.54%)	2,646 (7.47%)	314 (1.18%)	2,261 (7.47%)
41–60	131,791	102,951 (59.26%)	9,996 (48.09%)	4,280 (16.11%)	14,564 (48.09%)
61–70	56,494	35,866 (20.64%)	4,288 (27.89%)	7,894 (29.71%)	8,446 (27.89%)
71–80	38,942	16,604 (9.56%)	3,240 (16.56%)	14,084 (53%)	5,014 (16.56%)
**Race**					
White	207,149	142,231 (81.87%)	16,455 (83.73%)	23,106 (86.96%)	25,357 (83.73%)
Black	22,030	15,262 (8.78%)	2,114 (8.52%)	2,074 (7.81%)	2,580 (8.52%)
Other	21,585	16,244 (9.35%)	1,601 (7.75%)	1,392 (5.24%)	2,348 (7.75%)
**Marital status**					
Married	160,206	115,200 (66.31%)	12,298 (63.9%)	13,355 (50.26%)	19,353 (63.9%)
Single	32,416	23,617 (13.59%)	2,808 (11.51%)	2,506 (9.43%)	3,485 (11.51%)
Divorced	58,142	34,920 (20.1%)	5,064 (24.59%)	10,711 (40.31%)	7,447 (24.59%)
**Laterality**					
Right	126,809	87,974 (50.64%)	10,261 (50.06%)	13,413 (50.48%)	15,161 (50.06%)
Left	123,955	85,763 (49.36%)	9,909 (49.94%)	13,159 (49.52%)	15,124 (49.94%)
**Location**					
Central portion	14,614	9,642 (5.55%)	1,376 (5.8%)	1,839 (6.92%)	1,757 (5.8%)
Upper-inner quadrant	26,492	18,529 (10.66%)	2,043 (10.86%)	2,632 (9.91%)	3,288 (10.86%)
Lower-inner quadrant	13,472	9,006 (5.18%)	1,142 (5.73%)	1,590 (5.98%)	1,734 (5.73%)
Upper-outer quadrant	93,876	65,550 (37.73%)	6,828 (38.26%)	9,912 (37.3%)	11,586 (38.26%)
Lower-outer quadrant	17,581	12,300 (7.08%)	1,409 (6.91%)	1,780 (6.7%)	2,092 (6.91%)
Other	84,729	58,710 (33.79%)	7,372 (32.45%)	8,819 (33.19%)	9,828 (32.45%)
**Histological type**					
IDC	196,061	136,173 (78.38%)	15,604 (78.1%)	20,631 (77.64%)	23,653 (78.1%)
ILC	16,219	11,295 (6.5%)	1,575 (5.52%)	1,676 (6.31%)	1,673 (5.52%)
Mixed	19,768	13,817 (7.95%)	1,769 (7.88%)	1,796 (6.76%)	2,386 (7.88%)
Other	18,716	12,452 (7.17%)	1,222 (8.5%)	2,469 (9.29%)	2,573 (8.5%)
**Grade**					
Well	44,593	30,982 (17.83%)	1,683 (19.78%)	5,937 (22.34%)	5,991 (19.78%)
Moderate	107,443	73,030 (42.03%)	9,082 (43.33%)	12,209 (45.95%)	13,122 (43.33%)
Poor	93,890	66,675 (38.38%)	8,913 (34.41%)	7,880 (29.66%)	10,422 (34.41%)
Undifferentiated	4,838	3,050 (1.76%)	492 (2.48%)	546 (2.05%)	750 (2.48%)
Stage ^*^					
I	87,312	54,392 (31.31%)	4,165 (48.54%)	14,054 (52.89%)	14,701 (48.54%)
II	126,028	94,712 (54.51%)	9,152 (40.71%)	9,834 (37.01%)	12,330 (40.71%)
III	37,424	24,633 (14.18%)	6,853 (10.74%)	2,684 (10.1%)	3,254 (10.74%)
**Surgery**					
BCS	135,904	95,767 (55.12%)	8,114 (40.23%)	13,354 (50.26%)	18,669 (61.64%)
Mastectomy	114,860	77,970 (44.88%)	12,056 (59.77%)	13,218 (49.74%)	11,616 (38.36%)
**HR**					
Negative	49,400	36,315 (20.9%)	3,128 (19.76%)	3,972 (14.95%)	5,985 (19.76%)
Positive	201,364	137,422 (79.1%)	17,042 (80.24%)	22,600 (85.05%)	24,300 (80.24%)
**Chemotherapy**					
With	120,333	74,077 (42.64%)	7,901 (57.73%)	20,871 (78.55%)	17,484 (57.73%)
Without	130,431	99,660 (57.36%)	12,269 (42.27%)	5,701 (21.45%)	12,801 (42.27%)
**Radiotherapy**					
With	113,100	76,559 (44.07%)	9,703 (41.6%)	14,238 (53.58%)	12,600 (41.6%)
Without	137,664	97,178 (55.93%)	10,467 (58.4%)	12,334 (46.42%)	17,685 (58.4%)

* *Stage classification according to the 8th edition of AJCC staging*.

*SPCs, Second primary cancers; IPBC, initial primary breast cancer; IDC, Invasive ductal carcinoma; ILC, Invasive lobular carcinoma; Mixed, mix of IDC and ILC; HR, Hormone receptor; BCS, Breast conserving surgery*.

We randomly divided entire cohort into two parts: a development cohort (188,073 patients) and a validation cohort (62,691 patients). Baseline characteristics, such as initial diagnosis age, race and treatment-related factor, were similarly distributed in the development and validation cohorts (Supplemental Table 1).

### Identifying Factors Associated With SPC Risk

The pre-specified variable selection process selected eight variables for inclusion in the multivariable Fine and Gray model: age at initial diagnosis, race, laterality, histology, stage, HR, chemotherapy, and radiotherapy. Except laterality, all variables were included in the final model. Compared to a reference age group of 20–40 years, survivors of an elderly age had substantially elevated risks of SPCs [subdistribution hazard ratio (SHR) of 1.206 (95% CI: 1.100–1.323; *P* < 0.001) in the 41–60 group, 1.648 (95% CI: 1.494–1.818; *P* < 0.001) in the 61–70 group, and 1.332 (95% CI: 1.197–1.482; *P* < 0.001) in the 71–80 group]. Black women survivors had slightly elevated risks of developing SPCs compared with white survivors (SHR = 1.101; 95% CI: 1.015–1.194; *P* = 0.021). Survivors with mixed histology had an increased risk (SHR = 1.092; 95% CI: 1.004–1.188; *P* = 0.039) compared with survivors with IDC. Increasing IPBC stage was also associated with decreased risks of SPCs, with an SHR of 0.945 (95% CI: 0.896–0.996; *P* = 0.034) for stage II and 0.814 (95% CI: 0.750–0.884; *P* < 0.001) for stage III. HR positive status had significantly decreased risks by 12.0% compared to HR negative status (SHR = 0.880; 95% CI: 0.829–0.933; *P* < 0.001). In the present study, survivors treated with radiotherapy had elevated risks of developing SPCs compared to unirradiated survivors (SHR = 1.161; 95% CI: 1.109–1.217; *P* < 0.001). Patients with chemotherapy had a modest decreasing risk of developing SPCs (SHR = 0.880; 95% CI: 0.832–0.931; *P* < 0.001; Table 2).

**Table 2:** Factors associated with development of second primary cancer risks.

	**SHR**	**95% CI**	***p***
**Age**			
20–40	ref		
41–60	1.206	1.100–1.323	<0.001
61–70	1.648	1.494–1.818	<0.001
71–80	1.332	1.197–1.482	<0.001
**Race**			
White	ref		
Black	1.101	1.015–1.194	0.021
Other	0.993	0.914–1.079	0.860
**Histological type**			
IDC	ref		
ILC	0.974	0.881–1.077	0.600
Mix	1.092	1.004–1.188	0.039
Other	0.942	0.867–1.024	0.160
Stage^*^			
I	ref		
II	0.945	0.896–0.996	0.034
III	0.814	0.750–0.884	<0.001
**HR**			
Negative	ref		
Positive	0.880	0.829–0.933	<0.001
**Chemotherapy**			
With	ref		
Without	0.88	0.832–0.931	<0.001
**Radiotherapy**			
With	ref		
Without	1.161	1.109–1.217	<0.001

*p values obtained from the multivariable Fine and Gray competing model*.

* *Stage classification according to the 8th edition of AJCC staging*.

*SHR, Subdistribution hazard ratio; IDC, Infiltrating duct carcinoma; ILC, Invasive lobular carcinoma; Mixed, mix of IDC and ILC; HR, Hormone receptor*.

### Comparisons of Treatment and HR Status Related Risk by Organ Sites

**Figure 2:**
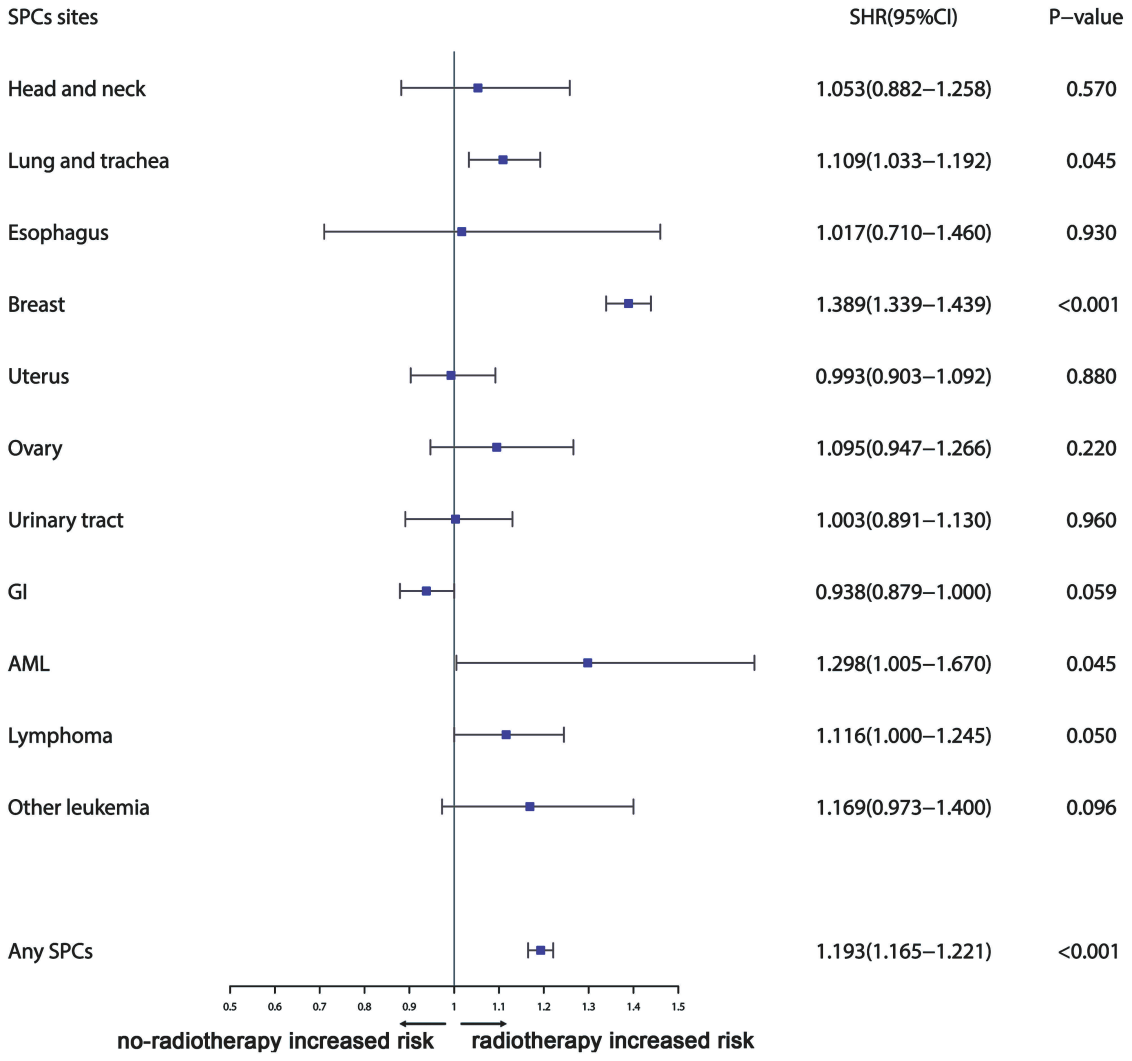
The forest plot comparing radiotherapy-related risk by selected organ sites. Head and neck: ICD-O codes C00-C14. Esophagus: ICD-O codes C15. Lung and trachea: ICD-O codes C33-C34. Breast: ICD-O codes C50. Uterus: ICD-O codes C54-C55. Ovary: ICD-O codes C56-C57. Urinary tract: ICD-O codes C63-C68. GI: Gastrointestinal, ICD-O codes C16-C26. AML: acute myeloid leukemia, ICD-O morphology codes 9860-9911. Other leukemia: ICD-O morphology codes 9912-9989. Lymphoma: ICD-O morphology codes: 9590-9837. SHR: Subdistribution hazard ratio. 95% CI: confidence interval.

**Figure 3:**
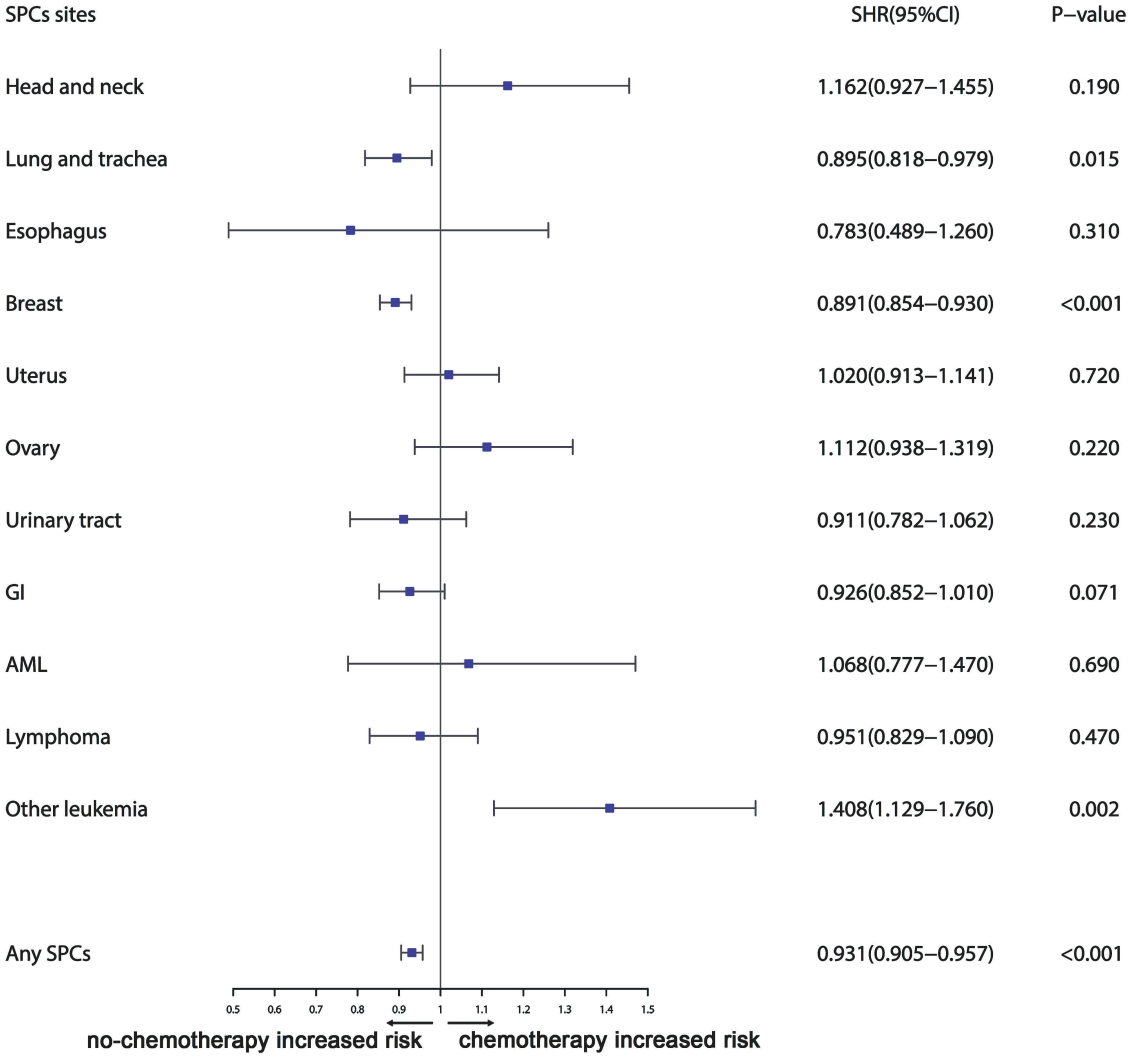
The forest plot comparing chemotherapy-related risk by selected organ sites. Head and neck: ICD-O codes C00-C14. Esophagus: ICD-O codes C15. Lung and trachea: ICD-O codes C33-C34. Breast: ICD-O codes C50. Uterus: ICD-O codes C54-C55. Ovary: ICD-O codes C56-C57. Urinary tract: ICD-O codes C63-C68. GI: Gastrointestinal, ICD-O codes C16-C26. AML: acute myeloid leukemia, ICD-O morphology codes 9860-9911. Other leukemia: ICD-O morphology codes 9912-9989. Lymphoma: ICD-O morphology codes: 9590-9837. SHR: subdistribution hazard ratio; 95% CI: confidence interval.

**Figure 4:**
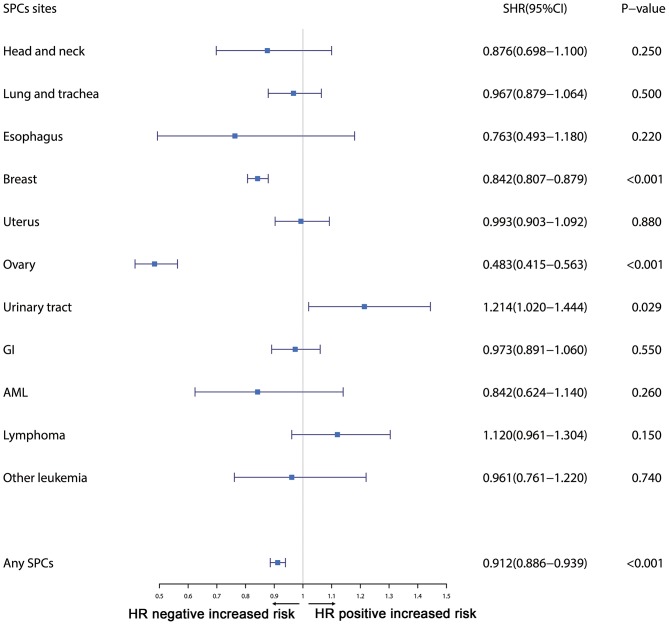
The forest plot comparing effect of HR status on second primary cancer risks by selected organ sites. Head and neck: ICD-O codes C00-C14. Esophagus: ICD-O codes C15. Lung and trachea: ICD-O codes C33-C34. Breast: ICD-O codes C50. Uterus: ICD-O codes C54-C55. Ovary: ICD-O codes C56-C57. Urinary tract: ICD-O codes C63-C68. GI: Gastrointestinal, ICD-O codes C16-C26. AML: acute myeloid leukemia, ICD-O morphology codes 9860-9911. Other leukemia: ICD-O morphology codes 9912-9989. Lymphoma: ICD-O morphology codes: 9590-9837. SHR: Subdistribution hazard ratio; 95% CI: confidence interval.

Furthermore, the effects of initial cancer-treatment (chemotherapy and radiotherapy) and HR status on the SPCs risk in selected organ sites were estimated based on the multivariable Fine and Gray risk model. We found that, after adjusting for age, race, histology, IPBC stage, HR, and chemotherapy, patients with radiotherapy had an elevated risk of any SPCs and with increased risks of lung cancer (SHR = 1.109; 95% CI: 1.033–1.192; *P* = 0.045), breast cancer (SHR = 1.389; 95% CI: 1.339–1.439; *P* < 0.001), and acute myeloid leukemia (AML) (SHR = 1.298; 95% CI: 1.005–1.670; *P* = 0.045). The results were shown in a forest plot (Figure 2). Patients with chemotherapy had a decreased risk of any SPCs and with decreased risks of lung (SHR = 0.895; 95% CI: 0.818–0.979; *P* = 0.015) and breast (SHR = 0.891; 95% CI: 0.854–0.930; *P* < 0.001) cancers, and with elevated risks of other leukemias (SHR = 1.408; 95% CI: 1.129–1.760; *P* = 0.002), after adjusting for age, race, histology, IPBC stage, HR, and radiotherapy. The results were shown in a forest plot (Figure 3). After adjusting for initial age of IPBC diagnosis, race, histology, IPBC stage, radiotherapy, and chemotherapy, HR-positive status patients had a declined risk of any SPCs and with decreased risks of second breast (SHR = 0.842; 95% CI: 0.807–0.879; *P* < 0.001) and ovarian cancers (SHR = 0.483; 95% CI: 0.415–0.563; *P* < 0.001), with elevated risks of urinary tract cancer (SHR = 1.214; 95% CI: 1.020–1.444; *P* = 0.029). The results were shown in a forest plot (Figure 4). The risk of developing SPCs by selected organ sites was summary in Table 3.

**Table 3:** Factors associated with development of second primary cancer risks by organ sites within the entire cohort.

	**Any SPC**	**Head and neck**	**Lung and trachea**	**Esophagus**	**Breast**	**Uterus**
**Variable**	**SHR (95% CI)**	**SHR (95% CI)**	**SHR (95% CI)**	**SHR (95% CI)**	**SHR (95% CI)**	**SHR (95% CI)**
**Age**						
20–40	Ref	Ref	Ref	Ref	Ref	Ref
41–60	1.121 (1.073–1.172)	1.525 (1.053–2.21)	4.808 (3.583–6.451)	1.593 (0.675–3.76)	0.754 (0.714–0.797)	3.48 (2.6–4.659)
61–70	1.475 (1.406–1.547)	1.747 (1.175–2.598)	10.05 (7.475–13.51)	2.296 (0.934–5.65)	0.662 (0.621–0.706)	4.391 (3.256–5.92)
71–80	1.200 (1.139–1.264)	1.681 (1.103–2.562)	7.183 (5.310–9.715)	2.651 (1.037–6.78)	0.434 (0.402–0.468)	3.574 (2.618–4.88)
**Race**						
White	Ref	Ref	Ref	Ref	Ref	Ref
Black	1.117 (1.072–1.164)	0.550 (0.36–0.841)	0.990 (0.866–1.133)	0.904 (0.453–1.8)	1.366 (1.291–1.446)	1.103 (0.931–1.306)
Other	0.950 (0.910–0.990)	1.106 (0.821–1.49)	0.649 (0.555–0.759)	0.782 (0.379–1.62)	1.079 (1.015–1.146)	1.111 (0.944–1.307)
**Histological type**						
IDC	Ref	Ref	Ref	Ref	Ref	Ref
ILC	0.983 (0.935–1.034)	1.072 (0.749–1.536)	0.937 (0.807–1.088)	1.276 (0.664–2.45)	1.009 (0.931–1.093)	0.802 (0.646–0.996)
Mix	1.065 (1.021–1.111)	0.939 (0.672–1.311)	0.902 (0.786–1.034)	0.717 (0.333–1.54)	1.217 (1.143–1.295)	0.944 (0.791–1.127)
Other	1.024 (0.983–1.067)	0.655 (0.446–0.962)	1.100 (0.974–1.242)	0.807 (0.408–1.6)	1.045 (0.983–1.112)	0.996 (0.84–1.181)
**Stage**						
I	Ref	Ref	Ref	Ref	Ref	Ref
II	0.939 (0.915–0.964)	0.970 (0.786–1.196)	0.991 (0.913–1.076)	1.169 (0.761–1.8)	0.881 (0.846–0.917)	1.007 (0.904–1.122)
III	0.802 (0.769–0.836)	0.943 (0.692–1.284)	0.930 (0.819–1.057)	1.428 (0.772–2.64)	0.651 (0.61–0.695)	0.898 (0.761–1.06)
**HR**						
Negative	Ref	Ref	Ref	Ref	Ref	Ref
Positive	0.912 (0.886–0.939)	0.876 (0.698–1.100)	0.967 (0.879–1.064)	0.763 (0.493–1.18)	0.842 (0.807–0.879)	1.009 (0.893–1.141)
**Radiotherapy**						
Without	Ref	Ref	Ref	Ref	Ref	Ref
With	1.193 (1.165–1.221)	1.053 (0.882–1.258)	1.109 (1.033–1.192)	1.017 (0.71–1.46)	1.389 (1.339–1.439)	0.993 (0.903–1.092)
**Chemotherapy**						
Without	Ref	Ref	Ref	Ref	Ref	Ref
With	0.931 (0.905–0.957)	1.162 (0.927–1.455)	0.895 (0.818–0.979)	0.783 (0.489–1.260)	0.891 (0.854–0.93)	1.02 (0.913–1.141)
	**Ovary**	**Urinary tract**	**GI**	**AML**	**Lymphoma**	**Other leukemia**
**Variable**	**SHR (95% CI)**	**SHR (95% CI)**	**SHR (95% CI)**	**SHR (95% CI)**	**SHR (95% CI)**	**SHR (95% CI)**
**Age**						
20–40	Ref	Ref	Ref	Ref	Ref	Ref
41–60	0.912 (0.718–1.158)	3.436 (2.234–5.283)	2.325 (1.900–2.840)	1.356 (0.791–2.330)	2.657 (1.865–3.784)	2.795 (1.517–5.15)
61–70	1.041 (0.798–1.358)	6.714 (4.338–10.39)	4.840 (3.943–5.940)	2.522 (1.445–4.400)	4.928 (3.436–7.066)	7.167 (3.877–13.25)
71–80	0.931 (0.694–1.249)	5.507 (3.517–8.622)	5.579 (4.528–6.880)	2.763 (1.501–5.090)	5.146 (3.562–7.436)	9.389 (5.022–17.56)
**Race**						
White	Ref	Ref	Ref	Ref	Ref	Ref
Black	0.499 (0.357–0.696)	0.965 (0.766–1.216)	1.206 (1.071–1.360)	1.032 (0.661–1.610)	0.895 (0.719–1.114)	1.004 (0.713–1.41)
Other	0.791 (0.601–1.041)	0.552 (0.416–0.732)	1.319 (1.182–1.470)	1.306 (0.878–1.940)	0.715 (0.567–0.901)	0.8 (0.551–1.16)
**Histological type**						
IDC	Ref	Ref	Ref	Ref	Ref	Ref
ILC	1.004 (0.726–1.388)	1.002 (0.788–1.274)	0.937 (0.816–1.080)	0.577 (0.306–1.090)	0.932 (0.74–1.173)	0.858 (0.583–1.26)
Mix	0.796 (0.582–1.088)	0.934 (0.748–1.166)	0.942 (0.831–1.070)	0.809 (0.490–1.330)	1.015 (0.833–1.236)	1.018 (0.73–1.42)
Other	0.864 (0.662–1.127)	0.822 (0.652–1.037)	0.980 (0.871–1.100)	1.138 (0.753–1.720)	0.938 (0.768–1.146)	1.101 (0.803–1.51)
**Stage**						
I	Ref	Ref	Ref	Ref	Ref	Ref
II	0.968 (0.823–1.14)	1.011 (0.881–1.16)	1.016 (0.942–1.090)	0.93 (0.684–1.260)	0.903 (0.798–1.023)	0.953 (0.775–1.17)
III	0.746 (0.575–0.967)	0.875 (0.702–1.09)	1.015 (0.904–1.140)	1.629 (1.101–2.410)	0.744 (0.606–0.913)	1.317 (0.998–1.74)
**HR**						
Negative	Ref	Ref	Ref	Ref	Ref	Ref
Positive	0.483 (0.415–0.563)	1.214 (1.02–1.444)	0.973 (0.891–1.060)	0.842 (0.624–1.140)	1.12 (0.961–1.304)	0.961 (0.761–1.22)
**Radiotherapy**						
Without	Ref	Ref	Ref	Ref	Ref	Ref
With	1.095 (0.947–1.266)	1.003 (0.891–1.13)	0.938 (0.879–1.000)	1.298 (1.005–1.670)	1.116 (1–1.245)	1.169 (0.973–1.4)
**Chemotherapy**						
Without	Ref	Ref	Ref	Ref	Ref	Ref
With	1.112 (0.938–1.319)	0.911 (0.782–1.062)	0.926 (0.852–1.010)	1.068 (0.777–1.470)	0.951 (0.829–1.09)	1.408 (1.129–1.76)

*p values obtained from the multivariable Fine and Gray competing model*.

*Head and neck: ICD-O codes C00-C14. Esophagus: ICD-O codes C15. Lung and trachea: ICD-O codes C33-C34. Breast: ICD-O codes C50. Uterus: ICD-O codes C54-C55. Ovary: ICD-O codes C56-C57. Urinary tract: ICD-O codes C63-C68. GI: ICD-O codes C16-C26. AML: acute myeloid leukemia, ICD-O morphology codes 9860-9911. Other leukemia: ICD-O morphology codes 9912-9989. Lymphoma: ICD-O morphology codes: 9590-9837*.

*Stage classification according to the 8th edition of AJCC staging*.

*SPCs, Second primary cancers; SHR, Subdistribution hazard ratio; IDC, Infiltrating duct carcinoma; ILC, Invasive lobular carcinoma; Mixed, mix of IDC and ILC; HR, Hormone receptor*.

### Establishment and Validation of the Competing Risks Nomogram

**Figure 5:**
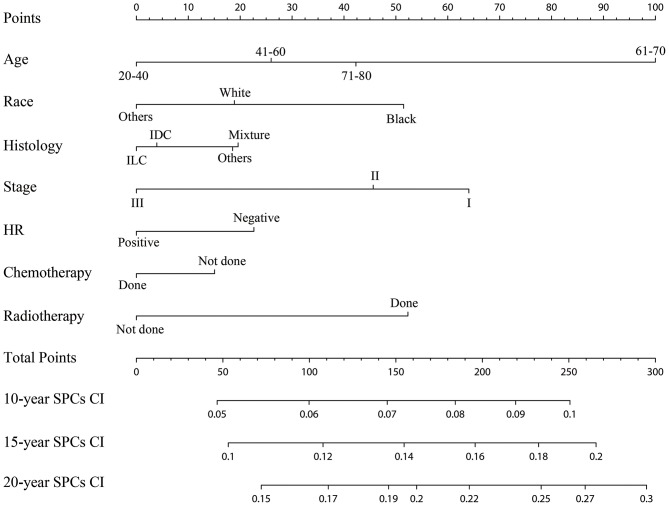
Competing risks nomogram for predicting the 10-, 15-, and 20-year risk of developing second primary cancers. The competing risks nomogram provides a method to calculate 10-, 15-, and 10-year probability of cumulative incidence (CI) of developing second primary cancers (SPCs) on the basis of a patient's combination of covariates. To use, locate the patient's age at initial diagnosis, draw a line straight up to the points axis to establish the score associated with that age. Repeat for the other five covariates (race, histology, stage, HR, chemotherapy, and radiotherapy). Add the score of each covariate together and locate the total score on the total points axis. Draw a line straight down to the 10-, 15-, and 20-year SPCs cumulative incidence axis to obtain the individual probability.

The established nomogram based on the multivariable Fine and Gray model shows the relative importance of each independent variable: age was the vital predictors of developing SPCs, followed by the IPBC stage, radiotherapy, race, HR status, histology, and chemotherapy (Figure 5). The validated C-index of this prediction model in the development cohort was 0.59 (95% CI: 0.56–0.61). The C-index in the validation cohort was 0.58 (95% CI: 0.55–0.62). Calibration plots for internal (development cohort) and external (validation cohort) validation of the prediction nomogram were shown in Supplemental Figure 2. Point assignment and risk score in the nomogram was summarized in Supplemental Table 2.

### Risk Stratification: Variation of SPC Risks Based on the Prediction Model

**Figure 6:**
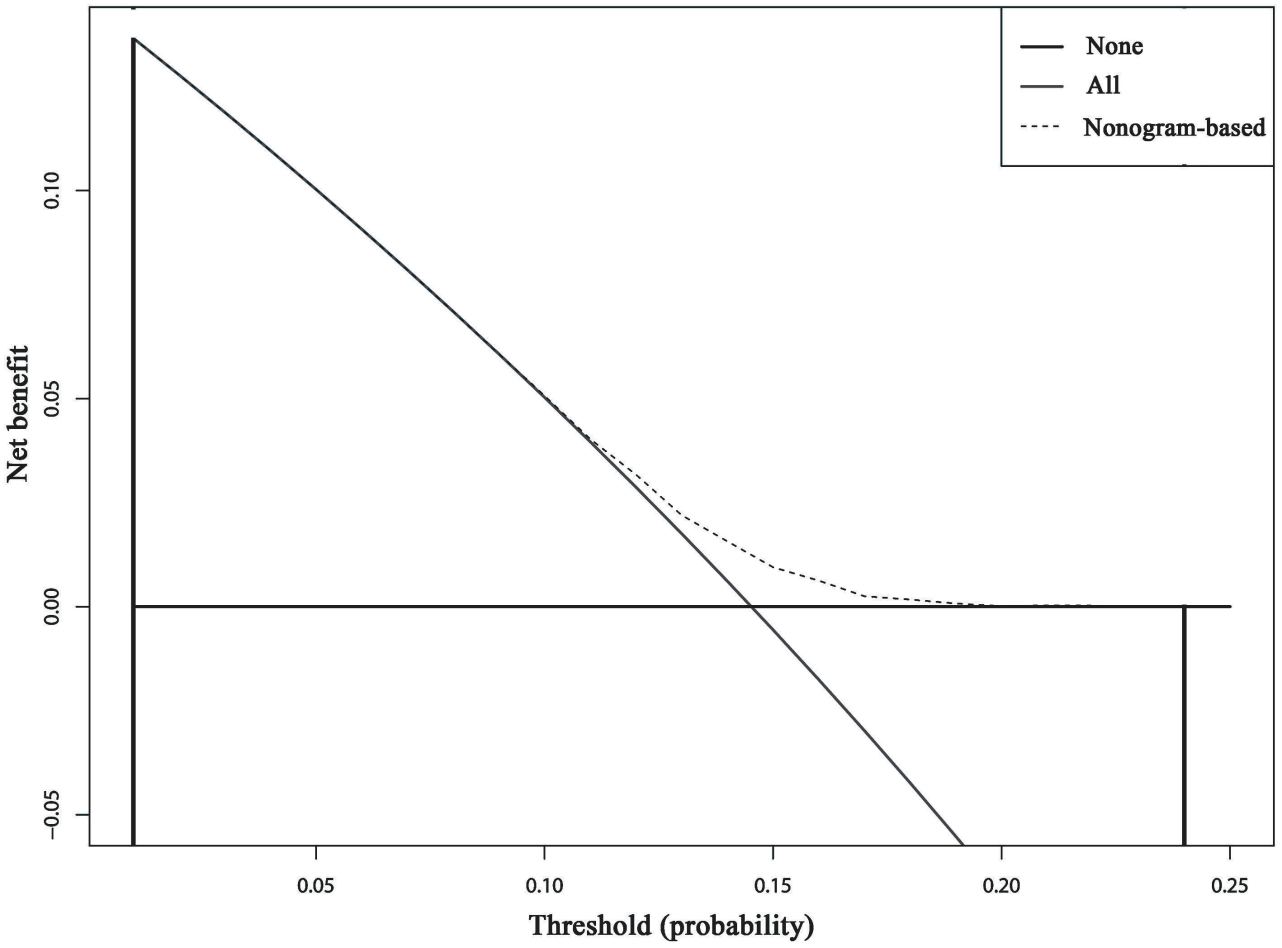
Decision curve analysis for the competing risks nomogram for 15-year second primary cancer risks in the validation cohort. The X-axis is the risk threshold probability that changes from 0 to 1 (right truncated at 0.25) and the Y-axis is the calculated net benefit for a given threshold probability. The dashed lines depict the net clinical benefit of the competing risks–based selection strategy for intervention, whereas the gray and black curves display the net benefits in the alternative strategies of treating all patients (gray) vs. treating no patients (black) in the cohort.

Cumulative incidence of developing SPCs across different risk subgroups defined nomogram-predicted risk score, which shows a wide stratification of the SPC risks at 15 years, from 12.01% for the 25th interquartile group to 17.42% for the 75th interquartile group with a statistical significance according to the Gray test (*P* < 0.001), demonstrated a well-discrimination among low and high risk subgroups (Supplemental Figure 3). The decision curve analysis using the 15-year risk of SPCs from the competing risks nomogram in the validation cohort to inform clinical decisions was better than the strategies of treat all or treat none across a wide range of thresholds between 0.01 and 0.24 (Figure 6).

## Discussion

In the present study, we calculate the cumulative incidence of SPCs among survivors of early-staged IPBC in the presence of competing events, evaluate risk factors for developing SPCs based on the multivariate Fine and Gray model, and build and externally validate a clinical prediction model. Our study supports and expands on previous studies demonstrating an elevated standardized incidence ratio (SIR) for SPCs following an IPBC, especially among elderly, early-stage, HR-negative, and irradiated survivors compared with the general population. To our knowledge, this is the first available nomogram for developing SPCs in IPBC survivors in the presence of competing events, which was helpful in individual risk estimation, patient follow-up and counseling. The DCA inform clinical decisions was better than the strategies of treat all or treat none across a wide range of thresholds between 0.01 and 0.24, which shows the higher clinical utility of our risk prediction model.

The previous studies demonstrated that young patients had a higher SIR than elderly patients (11). Inconsistent with those studies, our study found that elderly survivors have higher risks of developing SPCs. Previous studies is not directly comparing SPCs rates between older and younger population. To calculate age-adjusted standardized rates, one must first have the age-specific rates of disease for each of the populations to be compared. Studies based on SIR analysis, which is obtained by dividing the observed number of cases of breast cancer by the “expected” number of cases (12). Additionally, a high SIR does not necessarily imply a high cancer burden, given that the expected incidence of second cancers may be low (13). Overall breast cancer incidence increases with age, so the difference between the observed and expected risks of developing SPCs in the elderly group will be lower (12). And more young patients have a higher risk of mortality from IPBC, preventing the development of SPCs (13). More young patients have a higher risk of mortality from IPBC, preventing the development of SPCs (14). In addition, SIR study was not enough to ascribe causation when several risk factors are implicated (5).

Few studies have explored the effect of the extent of the initial disease on the development of SPCs. We found that increased patients with higher IPBC stage had a declined risk of SPCs, necessarily attributed to higher possibility of mortality from IPBC before SPCs occur (15). Consistent with previous studies, our study found that patients with HR-positive breast cancer had a declined SPCs risk. Of note, 60–90% of germline mutation BRCA1-associated breast cancers are HR negative, which may be a possible explanation for the increased second SPCs in HR-negative IPBC patients (16). BRCA1 and BRCA2 mutation patients had a respective 4.5- and 3.4-fold elevated risk of developing contralateral breast cancer (17). Previous studies found that Endocrine therapy approximately reduces 33% second breast cancer (18). We found that HR-positive patients were associated with an increased risk of second urinary tract cancers, which may be explained by hormone use. A Dutch study also found that hormonal therapy and shared etiological risk factors were associated with elevated risks of developing second urinary tract cancers (13).

In the present study, we compared treatment-related SPC risks by selected organ sites. A study estimated that 9% of any SPCs and 25% of the irradiation-associated site SPCs were ascribed to radiation therapy (19). A meta-analysis demonstrated that breast cancer patients with radiotherapy had an elevated overall risks of second non-breast cancer (20). In the present study, we found that patients with radiotherapy had an elevated risk of any SPCs and with elevated risks of lung, breast, and AML, which was consistent with the previous study. A study based on a SEER dataset demonstrated the risk of secondary malignancies and concluded that SPCs were significantly higher for cases that received chemotherapy after adjusting for known confounders (21). A population-based study including 58,068 Dutch patients demonstrated that patients with chemotherapy had a decreased risks of developing second non-breast cancers and colon and lung cancer (13). Our result was consistent with the Dutch study, finding that chemotherapy was associated with a modest protective effect of developing SPCs. Organ specific analysis showed that patients with chemotherapy had an elevated risks of leukemia (excluding AML). Given a SEER chemotherapy sensitivity of only 68% (22), our results should be treated with caution and need to be further confirmed in other population-based datasets.

A previous study also identified that black breast cancer survivors had a higher risk of developing SPCs (23). SPCs reflect not only the late effects of cancer and its treatment but also the influence of shared lifestyle, genetic susceptibility, environmental exposures, and gene-environment interactions (3). A Spanish cohort study demonstrated that smoking history, obesity, and high blood pressure were risk factors for SPCs (24). SEER does not provide all the above-listed information, which may lead to the lower C-index observed in our prediction model. Despite the lower C-index, our competing nomogram has a stratification ability to classify the cohort into subgroups with distinct risks of SPC development. SEER does not provide information of regimens. We recognize that the treatment regimens data is an inevitable limitation of our study.

Cumulative incidence of developing SPCs elevated over time and did not plateau. There is a significant difference in OS between survivors with and without SPCs. Consistent with previous studies, our study found that HR negative with radiotherapy and black race were significantly associated with increased risks of SPCs. In contrast, chemotherapy was associated with a modest protective effect. Inconsistent with previous reports, we found that elderly patients was associated with an elevated risk of developing SPCs. For the first time, we found that lower IPBC stage was also associated with elevated risk of developing SPCs. Furthermore, an externally validated clinical prediction model was established to help select high-risk patients.

## Data Availability Statement

The datasets generated for this study are available on request to the corresponding author.

## Author Contributions

DL conceived and designed the experiments, performed the experiments, analyzed the data, contributed reagents, materials and analysis tools, prepared figures and/or tables, and authored or reviewed drafts of the paper. SW and XT conceived and designed the experiments, performed the experiments, analyzed the data. CZ, NZ, YC, and DX performed the experiments and authored or reviewed drafts of the paper. YY conceived and designed the experiments, performed the experiments, authored or reviewed drafts of the paper, and approved the final draft.

### Conflict of Interest

The authors declare that the research was conducted in the absence of any commercial or financial relationships that could be construed as a potential conflict of interest.

## Supplementary Material

Supplemental Figure 1 The flow chart of the detailed inclusion and exclusion criteria.

Supplemental Figure 2 Internal (**A**: development cohort) and external (**B**: validation cohort) validation plots of the competing risks nomogram. The X-axis is average predicted probabilities of the competing risks nomogram. The Y-axis is the observed cumulative incidence probabilities for the respective cohort. Vertical lines are 95% CIs of the cumulative incidence. Dashed lines are the reference lines, which indicate where an ideal nomogram would lie.

Supplemental Figure 3 Cumulative incidence of second primary cancers (SPCs) by different risk subgroups defined by the estimated nomogram-predicted risk score. The marginal cumulative incidence of SPCs was calculated, and the difference of the cumulative incidences across distinct risk subgroups was tested using the Gray method.

Supplemental Table 1 Comparisons of patient characteristics of the study population in the development and validation cohorts.

Supplemental Table 2 Point assignment and risk score in the nomogram.
