# Supplementary figures and images for: Risk of Second Primary Cancers Among Long-Term Survivors of Breast Cancer
Source: Front Oncol. 2020 Jan 13;9:1426. doi: 10.3389/fonc.2019.01426 (PMC6970432; doi:10.3389/fonc.2019.01426)

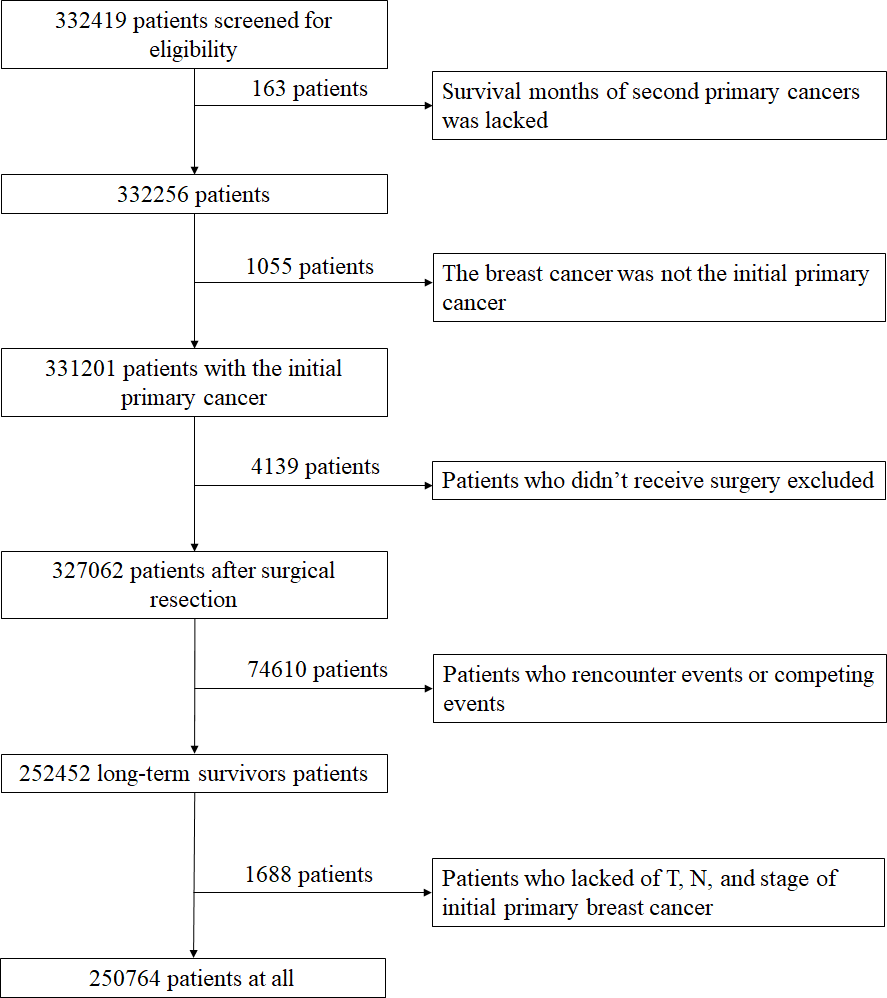

Supplement: Supplemental Figure 1 — The flow chart of the detailed inclusion and exclusion criteria. [file Image_1.TIF]

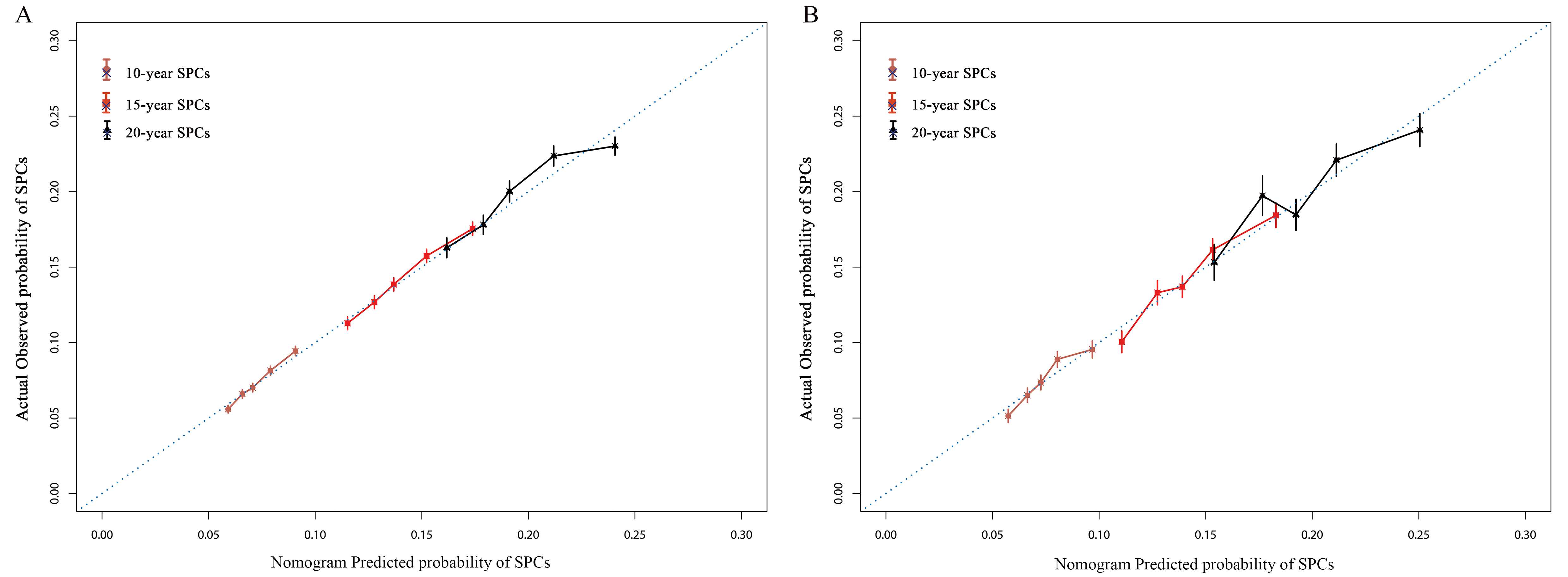

Supplement: Supplemental Figure 2 — Internal (A: development cohort) and external (B: validation cohort) validation plots of the competing risks nomogram. The X-axis is average predicted probabilities of the competing risks nomogram. The Y-axis is the observed cumulative incidence probabilities for the respective cohort. Vertical lines are 95% CIs of the cumulative incidence. Dashed lines are the reference lines, which indicate where an ideal nomogram would lie. [file Image_2.TIF]

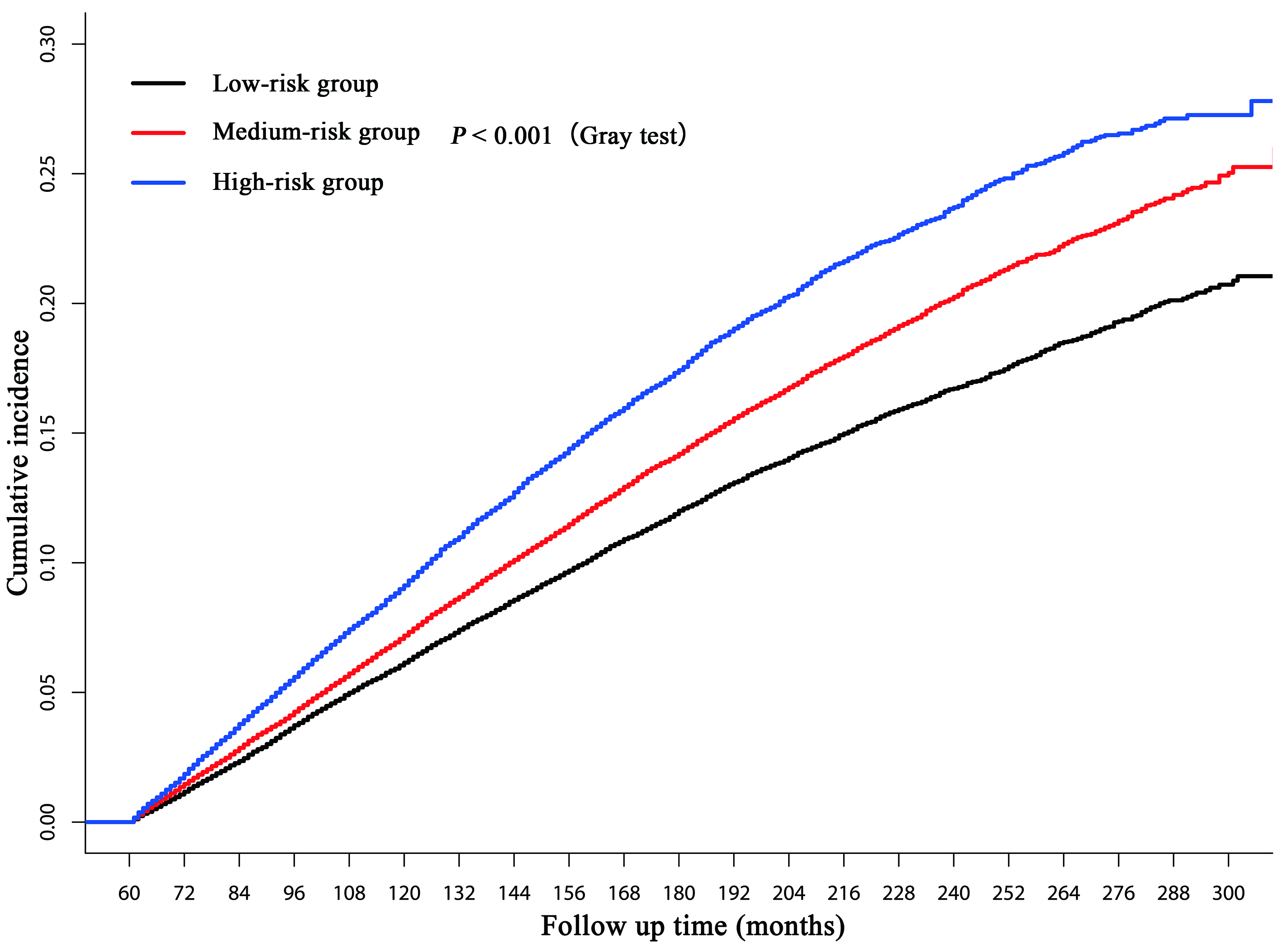

Supplement: Supplemental Figure 3 — Cumulative incidence of second primary cancers (SPCs) by different risk subgroups defined by the estimated nomogram-predicted risk score. The marginal cumulative incidence of SPCs was calculated, and the difference of the cumulative incidences across distinct risk subgroups was tested using the Gray method. [file Image_3.TIF]
