# Supplementary material for: Risk of Second Primary Cancers Among Long-Term Survivors of Breast Cancer
Source: Front Oncol. 2020 Jan 13;9:1426. doi: 10.3389/fonc.2019.01426 (PMC6970432; doi:10.3389/fonc.2019.01426)
Supplement: Supplemental Table 1 — Comparisons of patient characteristics of the study population in the development and validation cohorts. [file Table_1.docx]

**Supplemental Table 1: Comparisons of patient characteristics of the study population in the development and validation cohorts**

|  | **Development cohort**  **(188,073)** | **Validation cohort**  **(62,691)** | **SMDs** |
| --- | --- | --- | --- |
| Age |  |  | 0.004 |
| 20-40 | 17,646(9.38%) | 5,891(9.40%) |  |
| 41-60 | 98,772(52.52%) | 33,019(52.67%) |  |
| 61-70 | 42,362(22.52%) | 14,132(22.54%) |  |
| 71-80 | 29,293(15.58%) | 9,649(15.39%) |  |
| Race |  |  | 0.002 |
| White | 155,465(82.66%) | 51,684(82.44%) |  |
| Black | 16,382(8.71%) | 5,648(9.01%) |  |
| Other | 16,226(8.63%) | 5,359(8.55%) |  |
| Marital status |  |  | 0.007 |
| Married | 120,012(63.81%) | 40,194(64.11%) |  |
| Single | 24,331(12.94%) | 8,085(12.9%) |  |
| Divorced | 43,730(23.25%) | 14,412(22.99%) |  |
| Laterality |  |  | 0.003 |
| Right | 95,180(50.61%) | 31,629(50.45%) |  |
| Left | 92,893(49.39%) | 31,062(49.55%) |  |
| Location |  |  | <0.001 |
| Central portion | 10,899(5.8%) | 3,715(5.93%) |  |
| Upper-inner quadrant | 19,880(10.57%) | 6,612(10.55%) |  |
| Lower-inner quadrant | 10,143(5.39%) | 3,329(5.31%) |  |
| Upper-outer quadrant | 70,455(37.46%) | 23,421(37.36%) |  |
| Lower-outer quadrant | 13,257(7.05%) | 4,324(6.9%) |  |
| Other | 63,439(33.73%) | 21,290(33.96%) |  |
| Histological type |  |  | 0.010 |
| IDC | 147,201(78.27%) | 48,860(77.94%) |  |
| ILC | 12,113(6.44%) | 4,106(6.55%) |  |
| Mixed | 14,818(7.88%) | 4,950(7.9%) |  |
| Other | 13,941(7.41%) | 4,775(7.62%) |  |
| Grade |  |  | 0.008 |
| Well | 33,347(17.73%) | 11,246(17.94%) |  |
| Moderate | 80,447(42.77%) | 26,996(43.06%) |  |
| Poor | 70,625(37.55%) | 23,265(37.11%) |  |
| Undifferentiated | 3,654(1.94%) | 1,184(1.89%) |  |
| Stage * |  |  | <0.001 |
| I | 65,416(34.78%) | 21,896(34.93%) |  |
| II | 94,651(50.33%) | 31,377(50.05%) |  |
| III | 28,006(14.89%) | 9,418(15.02%) |  |
| HR |  |  | 0.001 |
| Negative | 37,024(19.69%) | 12,376(19.74%) |  |
| Positive | 151,049(80.31%) | 50,315(80.26%) |  |
| Surgery |  |  | 0.002 |
| BCS | 101,966(54.22%) | 33,938(54.14%) |  |
| Mastectomy | 86,107(45.78%) | 28753(45.86%) |  |
| Chemotherapy |  |  | 0.003 |
| With | 90,187(47.95%) | 30,146(48.09%) |  |
| Without | 97,886(52.05%) | 32,545(51.91%) |  |
| Radiotherapy |  |  | 0.002 |
| With | 84,781(45.08%) | 28,319(45.17%) |  |
| Without | 103,292(54.9%) | 34,372(54.83%) |  |

SMDs: Standardized mean differences, we considered SMDs of greater than 0.1 to indicate a potentially relevant difference between development and validation cohorts.

*Stage classification according to the 8th edition of AJCC staging.

Abbreviations: IDC: Invasive ductal carcinoma; ILC: Invasive lobular carcinoma; Mixed: mix of IDC and ILC; HR: Hormone receptor.
