# Supplementary material for: Risk of Second Primary Cancers Among Long-Term Survivors of Breast Cancer
Source: Front Oncol. 2020 Jan 13;9:1426. doi: 10.3389/fonc.2019.01426 (PMC6970432; doi:10.3389/fonc.2019.01426)
Supplement: Supplemental Table 2 — Point assignment and risk score in the nomogram. [file Table_2.docx]

**Supplemental Table 2:** **Point assignment and risk score in the nomogram**

| **Variables** | **Risk score** |
| --- | --- |
| Age |  |
| 20-40 | 0 |
| 41-60 | 26 |
| 61-70 | 100 |
| 71-80 | 42 |
| Race |  |
| White | 19 |
| Black | 51 |
| Other | 0 |
| Histological type |  |
| IDC | 4 |
| ILC | 0 |
| Mixed | 20 |
| Other | 19 |
| Stage * |  |
| I | 64 |
| II | 46 |
| III | 0 |
| HR |  |
| Negative | 23 |
| Positive | 0 |
| Chemotherapy |  |
| With | 15 |
| Without | 0 |
| Radiotherapy |  |
| With | 0 |
| Without | 52 |
| Total points | 10-year SPCs CI |
| 47 | 0.05 |
| 100 | 0.06 |
| 145 | 0.07 |
| 184 | 0.08 |
| 219 | 0.09 |
| 251 | 0.10 |
| Total points | 15-year SPCs CI |
| 53 | 0.10 |
| 108 | 0.12 |
| 155 | 0.14 |
| 196 | 0.16 |
| 233 | 0.18 |
| 266 | 0.20 |
| Total points | 20-year SPCs CI |
| 72 | 0.15 |
| 111 | 0.17 |
| 146 | 0.19 |
| 162 | 0.20 |
| 192 | 0.22 |
| 234 | 0.25 |
| 259 | 0.27 |
| 295 | 0.30 |

Risk score values obtained from the competing nomogram based on the multivariable Fine and Gray competing model.

* Stage classification according to the 8th edition of AJCC staging.

Abbreviations: SPCs: Second primary cancers; CI: Cumulative incidence; IDC: Infiltrating duct carcinoma; ITC: Invasive lobular carcinoma; Mixed: mix of IDC and ILC; HR: Hormone receptor;
